# Aligning cardiac monitoring with American Heart Association Guidelines: Impact on utilization, hemodynamic monitoring, and outcomes

**DOI:** 10.1371/journal.pone.0338943

**Published:** 2026-01-30

**Authors:** Brian Hilliard, Tanvi Mehta, James Grace, Deborah L. Pestka, Nicholas E. Ingraham, Timothy Beebe, Christopher Tignanelli, Genevieve B. Melton, Nathan Shippee, Joseph S. Koopmeiners, Michael Usher

**Affiliations:** 1 Department of Medicine, University of Minnesota Medical School, Minneapolis, Minnesota, United States of America; 2 Division of Biostatistics, School of Public Health, University of Minnesota, Minneapolis, Minnesota, United States of America; 3 Department of Surgery, University of Minnesota Medical School, Minneapolis, Minnesota, United States of America; 4 Center for Learning Health System Sciences, University of Minnesota Medical School, Minneapolis, Minnesota, United States of America; 5 Division of Health Policy Management, School of Public Health, University of Minnesota, Minneapolis, Minnesota, United States of America; 6 Institute for Health Informatics, University of Minnesota, Minneapolis, Minnesota, United States of America; Scuola Superiore Sant'Anna, ITALY

## Abstract

**Background:**

Overuse of continuous cardiac monitoring can lead to poor patient experience, increased costs, and decreased efficiency. Because significant variation in continuous cardiac monitoring ordering exists, implementation strategies that promote care in alignment with practice standards and an examination of use cases that fall outside of standards are needed. The purpose of this study, therefore, was to evaluate if implementation of American Heart Association (AHA) practice standards on continuous cardiac monitoring could reduce utilization without jeopardizing patient safety.

**Methods:**

We conducted a prospective pre-post study including a 2 year prospectively collected baseline against a 10-month post intervention period within a 10-hospital health system. An electronic health record (EHR) order set was implemented to align care with AHA continuous cardiac monitoring practice standards. We compared continuous cardiac monitoring utilization, adherence to standards, as well as clinical outcomes including mortality and length of stay. Finally, we investigated the rate and impact of hemodynamically significant events (hypotension, bradycardia, and tachycardia) before and after the intervention.

**Results:**

We compared 117,814 hospitalizations pre-implementation against 49,006 post implementation finding significant reductions in total telemetry use, and no significant change in outcomes. Overall, patients with telemetry use outside of standards had higher mortality, longer length of stay, and higher readmission rates. The intervention was associated with a higher rate of hypotensive events which occurred off cardiac monitoring. This was not associated with worse outcomes.

**Conclusions:**

An EHR tool to align care with continuous cardiac monitoring practice standards safely reduced overall continuous cardiac monitoring utilization. Use outside of practice standards persisted and was primarily focused on monitoring for potential hemodynamic instability. We found no evidence that continuous cardiac monitoring was associated with improved outcomes in unstable patients. Continuous cardiac monitoring for potentially unstable patients can likely be replaced for non-cardiac indications with continuous heart rate monitoring.

## Introduction

Continuous cardiac monitoring and remote telemetry utilization has steadily increased since its inception in the 1960s from the intensive care unit (ICU) to non-critical care settings [1]. The central purpose of continuous cardiac monitoring is to assist with recognition of clinically significant cardiac arrhythmias to support diagnosis, prevention, and rapid interventions [2]. However, cardiac monitoring continues to be used frequently for indications beyond monitoring for cardiac arrhythmias outside of the ICU [3].

Importantly, this potential overuse has significant downsides. First, continuous cardiac monitoring requires specific equipment and training, creating a bottleneck in the admission process, and can lead to prolonged emergency department (ED) wait times and length of stay [4]. Furthermore, telemetry can directly negatively impact patient care. For example, alarm fatigue and care disruptions caused by telemetry can frustrate and overburden nursing teams. In addition, patients may be negatively impacted by increased tethering, unnecessary anxiety around continuous monitoring, discomfort with patches/adhesives, and sleep interruption caused by continuous cardiac monitoring [3,5,6]. Finally, among patients who lack clear benefit, continuous cardiac monitoring alarms rarely result in changes in care [7].

Widespread, but inconsistent, use of continuous cardiac monitoring has led to the development of practice standards by the American Heart Association (AHA) [1,8]. Studies following the development of the AHA practice standards showed that as many as 43% of monitored patients lacked a recommended indication for monitoring [9]. Another study showed that 19.1% of indications were appropriate according to AHA practice standards, 20.7% were questionable, and 60.1% were not indicated [10]. Consequently, despite the existence of practice standards, continuous cardiac monitoring remains overused with medical teams often unaware that their patients are being monitored [4].

Although consensus practice standards and widely distributed public campaigns to reduce continuous cardiac monitoring overuse, such as *Choosing Wisely* [11], continuous cardiac monitoring remains overused in many settings. Furthermore, several gaps in the continuous cardiac monitoring evidence base remain [12]. Chief among them is the utility of continuous cardiac monitoring in general medicine patients with potential hemodynamic instability, such as a gastrointestinal bleed or sepsis. While continuous cardiac monitoring may allow higher resolution monitoring of heart rate, it is unclear whether this promotes more prompt responses to hemodynamic changes and better outcomes. In addition, it is unknown if restricting continuous cardiac monitoring duration for potentially high-risk patients results in worse patient outcomes [6]. Therefore, the objectives of this study were to (1) evaluate if embedding AHA continuous cardiac monitoring practice standards into routine ordering could reduce inappropriate use, and (2) evaluate the impact of continuous cardiac monitoring use and outcomes for patients where monitoring did not follow the AHA practice standards.

## Methods

### Setting and study design

To measure the impact of alignment of continuous cardiac monitoring with AHA guidelines, we conducted a practice embedded pre-post study at a 10-hospital health system in the upper Midwest that includes multiple community hospitals and a single large academic tertiary referral center. This includes multiple units for continuous cardiac monitoring, remote cardiac monitoring, and ICUs. Pre-intervention, there was no standardized mandate for reporting cardiac monitoring indications, and no standardized guidance for duration for use. This study was approved by the University of Minnesota Institutional Review Board (Study #00014799). As this was a quality improvement project, informed consent from ordering providers was not required nor obtained.

### Population

Patients were included if they were older than 18, admitted to the hospital under inpatient or observation status, and had at least a 24-hour stay during the study period. Additionally, we excluded patients who opted out of clinical research for any reason during the study period. Patients who were admitted during the week of implementation, or patients who were still admitted at study completion, were additionally excluded.

### Intervention

A CDS tool was developed within the EHR to promote adherence to AHA practice standards for continuous cardiac monitoring in both initiation and duration. In collaboration with system collaborators, including cardiology, cardiothoracic surgery, and internal medicine clinical leadership, the AHA practice standards were condensed into a series of large categories and then focused on indications with recommended durations (Table 1). Providers were additionally given the option of an “Other” category and prompted to write in the requested indication. This tool was followed by an interruptive best practice alert (BPA) at the completion of the recommended duration. At that time, providers would be prompted to re-order cardiac monitoring if they felt it was warranted. This study was designed as a pre-post intervention comparing 2 years of a retrospectively historical baseline against 6 months post implementation. Data collection began 1/21/2020, the intervention was initiated 1/21/2022 and continued until 11/31/2022. The authors had access to data that could identify individual providers.

**Table 1:** Patient demographics and outcomes pre and post implementation stratified by whether they had a continuous cardiac monitoring order during their stay.

	Pre-Implementation		Post Implementation	
	No Continuous cardiac monitoring (N = 63570)	Continuous cardiac monitoring (N = 54244)	No Continuous cardiac monitoring (N = 30232)	Continuous cardiac monitoring (N = 18774)
**Demographics**				
Age (years), Mean (SD)	43.24 (24.7)	62.16 (21.1)	46.98 (23.3)	64.40 (24.7)
Male, n (%)	19464 (30.6%)	26233 (48.4%)	9150 (30.3%)	9424 (50.2%)
White, n (%)	46451 (73.1%)	43929 (81.0%)	22531 (74.5%)	15299 (81.5%)
Black, n (%)	5929 (9.3%)	4186 (7.7%)	2892 (9.6%)	1484 (7.9%)
Native, n (%)	887 (1.4%)	737 (1.4%)	378 (1.3%)	232 (1.2%)
Asian, n (%)	6429 (10.1%)	3146 (5.8%)	2592 (8.6%)	1037 (5.5%)
Other Race, n (%)	3874 (6.1%)	2246 (4.1%)	1839 (6.1%)	722 (3.8%)
Hispanic, n (%)	1519 (2.4%)	801 (1.5%)	1110 (3.7%)	409 (2.2%)
**Comorbidities**				
Congestive heart failure, n (%)	4340 (6.8%)	13753 (25.4%)	2494 (8.2%)	5942 (31.7%)
Hypertension, n (%)	21867 (34.4%)	34602 (63.8%)	12123 (40.1%)	13285 (70.8%)
Chronic kidney disease, n (%)	6490 (10.2%)	8746 (16.1%)	4381 (14.5%)	4714 (25.1%)
Diabetes, n (%)	9764 (15.4%)	16428 (30.3%)	5385 (17.8%)	6448 (34.3%)
**Hemodynamic events**				
Tachycardia, n (%)	1907 (3.0%)	1195 (2.2%)	777 (2.6%)	444 (2.4%)
Tachycardia off Continuous cardiac monitoring n (%)	1844 (2.9%)	311 (0.6%)	764 (2.5%)	118 (0.6%)
Bradycardia, n (%)	187 (0.3%)	818 (1.5%)	102 (0.3%)	279 (1.5%)
Bradycardia off Continuous cardiac monitoring n (%)	179 (0.3%)	242 (0.4%)	99 (0.3%)	88 (0.5%)
Hypotensive, n (%)	7160 (11.3%)	13137 (24.2%)	3553 (11.8%)	4751 (25.3%)
Hypotension off Continuous cardiac monitoring n (%)	6922 (10.9%)	4339 (8.0%)	3482 (11.5%)	1773 (9.4%)
**Outcomes**				
Length of Stay, (days) Mean (SD)	4.134 (9.3)	5.876 (8.2)	4.308 (8.23)	6.222 (8.23)
30- Day Readmission, n (%)	3969 (6.2%)	5216 (9.6%)	2216 (7.3%)	2113 (11.3%)
In-Hospital Mortality, n (%)	566 (0.9%)	1808 (3.3%)	225 (0.7%)	601 (3.2%)
30-Day Mortality, n (%)	1387 (2.2%)	3657 (6.7%)	642 (2.1%)	1325 (7.1%)

### Measures and outcomes

All measures were directly extracted from the EHR with 50 randomly selected charts manually validated. The primary measure was the total number of days with continuous cardiac monitoring per hospital admission within the first 10 days of admission. Secondary measures included the proportion of admissions with any cardiac monitoring, the duration of monitoring when applied, the proportion of telemetry orders that had an indication reported, and proportion of monitoring which was in excess of AHA guidelines. We collected patient demographics, chronic comorbidities by Elixhauser, admission diagnosis by ICD-10 code and MS-DRG code, and procedure related cardiac monitoring orders [13,14]. We additionally measured length of hospital stay, ICU transfer rates, in-hospital mortality, 30-day all-cause mortality, and 30-day readmission rates. All data both before and after intervention. was collected prospectively prior to intervention using standardized formatting.

We assessed the impact of the intervention on the rate of capture of hemodynamically significant events and subsequent outcomes. This included patients with two consecutive observations of bradycardia (heart rate (HR) < 40), tachycardia (HR > 150), and hypotension (mean arterial pressure < 65). These thresholds were chosen as common triggers for urgent management and delayed recognition could result in major complications such as stroke, myocardial infarction, and acute kidney injury.

### Statistics

Our analysis focused on comparing continuous cardiac monitoring utilization pre and post intervention, as well as clinical outcomes and adverse outcomes on and off continuous cardiac monitoring both pre and post intervention. We provide summary statistics for patient demographics and clinical characteristics on and off continuous cardiac monitoring both pre and post intervention. Continuous covariates were summarized by the sample mean and standard deviation, while categorical covariates were summarized by counts and the sample proportion. We estimated the effect of the intervention on continuous cardiac monitoring, hemodynamic events, and clinical outcomes via an interrupted time series model fit using generalized estimating equations (GEE) with a working independence correlation structure and implemented via the geepack package in R [15,16]. We fit both an unadjusted model and a model adjusted for age, sex, race, ethnicity, and Elixhauser comorbidities. For this analysis, our primary focus was on the impact of the intervention on the intercept because we hypothesized that the impact of the CDS would be immediate and sustained. The interaction with the slope is also reported, but not a focus of this study.

To evaluate the impact of hemodynamic events off continuous cardiac monitoring, we fit logistic regression models by day for patients who had a hypotensive episode or arrhythmia adjusting for patient age, race, ethnicity, sex, and Elixhauser comorbidities with outcomes of 30-day readmission and mortality. We adjusted by hospital day, as we observed crude differences between the day of the event and outcome, and hypothesized that our intervention may impact the type of patient with continuous cardiac monitoring beyond the first two hospital days. We compared the odds ratios of those who had a hypotensive episode off continuous cardiac monitoring to those on before and after implementation of the CDS. For patients with multiple hospital admissions, we randomly selected hospitalization. We report pre-CDS and post-CDS odds ratios with 95% confidence intervals by day for both outcomes, 30-day readmission and mortality.

We performed several sensitivity analyses, such as excluding peri-procedural telemetry orders, patients initially admitted into the ICU (where continuous cardiac monitoring is standard practice but may not be ordered), and patients with or without continuous cardiac monitoring indications, across multiple hospitals in the system, and by race in accordance with best practices [17]. Initially, since COVID-19 could confound both use and outcomes for continuous cardiac monitoring, we determined if our findings remained robust with and without COVID-19 admissions [18]. Data processing of raw EHR data was performed in STATA (v17.0 Statacorp). Final statistical analysis was performed with R while Prism (v7, Graphpad) was used to visualize summary data [19].

## Results

Of the patients that were admitted during the pre-implementation phase, 54,244 (46.0%) were admitted with continuous cardiac monitoring at some time during their inpatient stay, while 63,570 (54.0%) did not have continuous cardiac monitoring ordered (Table 1). On average, patients who were observed with continuous cardiac monitoring were older (62.2 vs 43.2, p < 0.001), more likely to be male (48.4% vs 30.6%, p < 0.001%), white (81.0% vs 73.1%, p < 0.001) and had higher rates of comorbidities, arrhythmias, hypotensive episodes, 30-day readmissions, and mortality.

Post implementation, 30,232 (61.7%) patients were admitted without continuous cardiac monitoring, and 18,774 (38.3%) were admitted with continuous cardiac monitoring at one point in their stay. Patients admitted with continuous cardiac monitoring were older (62.2 vs 64.4, p < 0.001). Post implementation, comorbidities were observed at a higher rate among patients on continuous cardiac monitoring (25.4% vs 31.7% for congestive heart failure, for example), and had an overall higher comorbidity index score.

**Fig 1:**
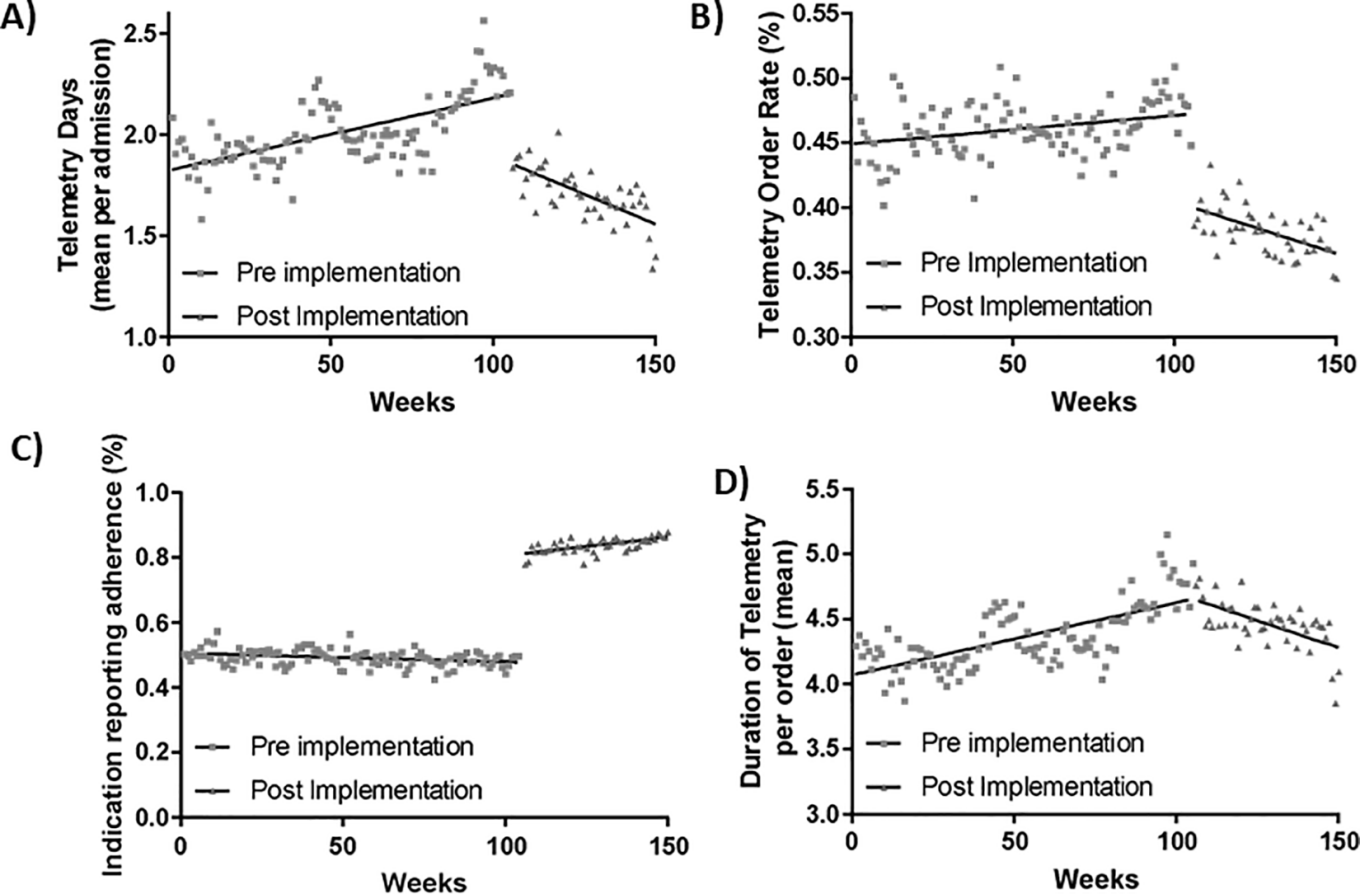
Trends in continuous cardiac monitoring utilization pre- and post- implementation. Unadjusted rate and linear trend pre- and post-intervention for the total number of continuous cardiac monitoring days per admission **(A)**, percentage of patients with any continuous cardiac monitoring order **(B)**, the proportion of continuous cardiac monitoring orders that adhered to the new process and reporting at least one indication **(C)**, and average duration of continuous cardiac monitoring use once ordered **(D)**.

Total continuous cardiac monitoring utilization and continuous cardiac monitoring order rates were significantly lower post-intervention vs. pre-intervention (Fig 1 and S5 Table in S1 File). These differences remained significant after adjusting for patient factors and temporal trends including total continuous cardiac monitoring days (p < 0.001), proportion of patients with continuous cardiac monitoring (p < 0.001), and the rate by which continuous cardiac monitoring had a recorded indication (p < 0.001) (Table 2). There was no significant difference in continuous cardiac monitoring duration post-intervention (p = 0.1).

**Table 2:** Impact of continuous cardiac monitoring CDS on process, hemodynamic monitoring and outcomes, measured by interrupted time series analysis.

	Intercept			Slope		
Measure	Treatment Effect*	95% CI	p-value	Treatment Effect*	95% CI	p-value
**Process**						
Total continuous cardiac monitoring days^1^	−0.3841	(−0.443 to −0.325)	<0.001	−0.0079	(−0.010 to −0.006)	<0.001
Continuous cardiac monitoring ordered^2^	0.6775	(0.645 to 0.712)	<0.001	0.9963	(0.994 to 0.998)	<0.001
Indication Reporting Adherence^2^	1.7176	(1.628 to 1.812)	<0.001	0.9996	(0.998 to 1.002)	0.7089
Continuous cardiac monitoring duration^1^	−0.076	(−0.167 to 0.015)	0.101	−0.0118	(−0.015 to −0.008)	<0.001
**Hemodynamic events**						
Tachycardia^2^	1.0606	(0.920 to 1.223)	0.4186	0.994	(0.989 to 1.000)	0.0388
Bradycardia^2^	0.7504	(0.579 to 0.972)	0.0297	1.0072	(0.997 to 1.017)	0.1555
Arrhythmia off continuous cardiac monitoring^2^	1.162	(0.995 to 1.357)	0.0574	0.998	(0.992 to 1.003)	0.4126
Hypotension^2^	1.0000	(0.942 to 1.062)	>0.99	0.9948	(0.993 to 0.997)	<0.001
Hypotension off continuous cardiac monitoring^2^	1.2072	(1.122 to 1.299)	<0.001	0.9978	(0.995 to 1.001)	0.115
**Outcomes**						
In Hospital Mortality^2^	0.8142	(0.691 to 0.959)	0.0138	0.9847	(0.978 to 0.991)	<0.001
30-Day Readmission^2^	1.0651	(0.985 to 1.152)	0.1148	0.9948	(0.992 to 0.998)	<0.001
Admission Delay^1^	0.2824	(−0.007 to 0.572)	0.0559	−0.0124	(−0.023 to −0.002)	0.025
Length of Stay^1^	−0.0754	(−0.267 to 0.116)	0.441	−0.0265	(−0.033 to −0.020)	<0.001
Log Length of Stay^1^	−0.0183	(−0.036 to 0.000)	0.0463	−0.0028	(−0.004 to −0.002)	<0.001

CI, confidence interval

*Treatment effects are mean differences for continuous outcomes (indicated by ^1^) and odds ratios for binary outcomes (indicated by ^2^). Confidence intervals are on the same scale as the treatment effects.

CDS implementation had no significant impact on length of stay, 30-day all cause readmission, in-hospital mortality, or 30-day all-cause mortality (Table 2 and S1 Fig in S1 File). We also observed no reduction in time from ED presentation to transfer to an inpatient bed with or without continuous cardiac monitoring.

We evaluated variations in continuous cardiac monitoring duration, and duration in excess of practice standards stratified by reported indication (Table 3). There were a total 84,009 continuous cardiac monitoring days post-intervention, of which 34,393 (29%) were in excess of AHA practice standards (S6 Table in S1 File). Excess utilization was widely spread across indications. For example, cardiac indications accounted for 34.1% of total utilization and 34.3% of excess utilization. Similarly, medical indications (such as severe electrolyte derangements) accounted for 10.7% of total continuous cardiac monitoring days and 9.6% of excess continuous cardiac monitoring days. Patients who were monitored on continuous cardiac monitoring in excess of AHA practice standards overall had higher mortality, length of stay, ICU transfer rates, and readmission rates (S7 Table in S1 File).

**Table 3:** Average continuous cardiac monitoring use and excess use by indication post implementation.

Indication	N	Total Continuous cardiac monitoring Days (mean, SD)	Excess Days (mean, SD)
**Cardiac**			
AMI (NSTEMI/ STEMI) (48 hours)	577	4.5 (2.5)	0.8 (2.1)
Acute decompensated heart failure (48 hours)	1890	5.3 (2.5)	1.8 (2.3)
Bradycardias (48 hours)	339	4.2 (2.2)	0.7 (2.0)
Chest pain/ ACS rule out (24 hours)	790	4.2 (2.4)	1.4 (2)
Infective endocarditis (48 hours) – additional guidance recommending until clinically stable	25	6.6 (2.8)	3.2 (2.7)
QTc prolonging medication (48 hours)	294	4.3 (2.2)	0.9 (2.0)
Syncope- high cardiac risk (48 hours)	207	4.5 (2.3)	1.1 (2.2)
Syncope- low cardiac risk (24 hours)	341	4.0 (2.1)	1.5 (1.9)
Tachyarrhythmias, acute (48 hours)	1755	4.7 (2.4)	1.2 (2.2)
**Cardiac procedure**			
Open heart surgery (72 hours)	324	7.6 (1.9)	1.8 (2.3)
Post- EP procedure (48 hours)	95	3.5 (2.4)	−0.06 (1.8)
Post- ICD or pacemaker placement (48 hours)	3	1.7 (0.6)	−1.3 (0.6)
Post- PCI/ percutaneous cardiac intervention (24 hours)	62	4.5 (2.8)	1.7 (2.2)
Post- PCI/Angiogram (24 hours)	351	4.1 (2.5)	1.2 (1.9)
Transcatheter structural interventions (24 hours)	211	2.7 (1.6)	0.4 (1.4)
**Medical**			
Drug overdose (24 hours)	100	3.8 (2.0)	1.4 (1.9)
Electrolyte Imbalance (24 hours)- Magnesium <1.3 mg/ml; Potassium < =2.8 or > 5.5 mg/ml	902	4.2 (2.3)	1.6 (2.1)
Stroke, acute (48 hours)	1043	4.0 (2.1)	0.6 (1.8)
Procedural area	851	2.6 (1.9)	−0.6 (1.7)
ICU	2022	5.2 (2.8)	1.6 (2.6)
Other	3545	4.2 (2.4)	1.5 (2.0)
Non-Adherent to Indication Reporting	3045	4.5 (2.6)	1.4 (2.5)

SD, standard deviation; AMI, acute myocardial infarction; NSTEMI, non-ST-segment elevation myocardial infarction; STEMI, ST-segment elevation myocardial infarction; ACS, acute coronary syndrome; EP, electrophysiology; ICD, implantable cardioverter defibrillator; PCI, percutaneous coronary intervention; ICU, intensive care unit.

A total of 17.7% of continuous cardiac monitoring use fell to indications outside of AHA practice standards, accounting for 21.3% of excess days. Non-AHA practice standard continuous cardiac monitoring utilization fell into multiple broad disease categories, including respiratory failure, sepsis, gastrointestinal bleed, diabetic ketoacidosis and alcohol withdrawal (S8 Table). Patients with non-AHA continuous cardiac monitoring orders had longer length of stay, higher rates of hypotension, ICU transfer, and mortality when compared to patients without continuous cardiac monitoring (S9 Table in S1 File).

**Fig 2:**
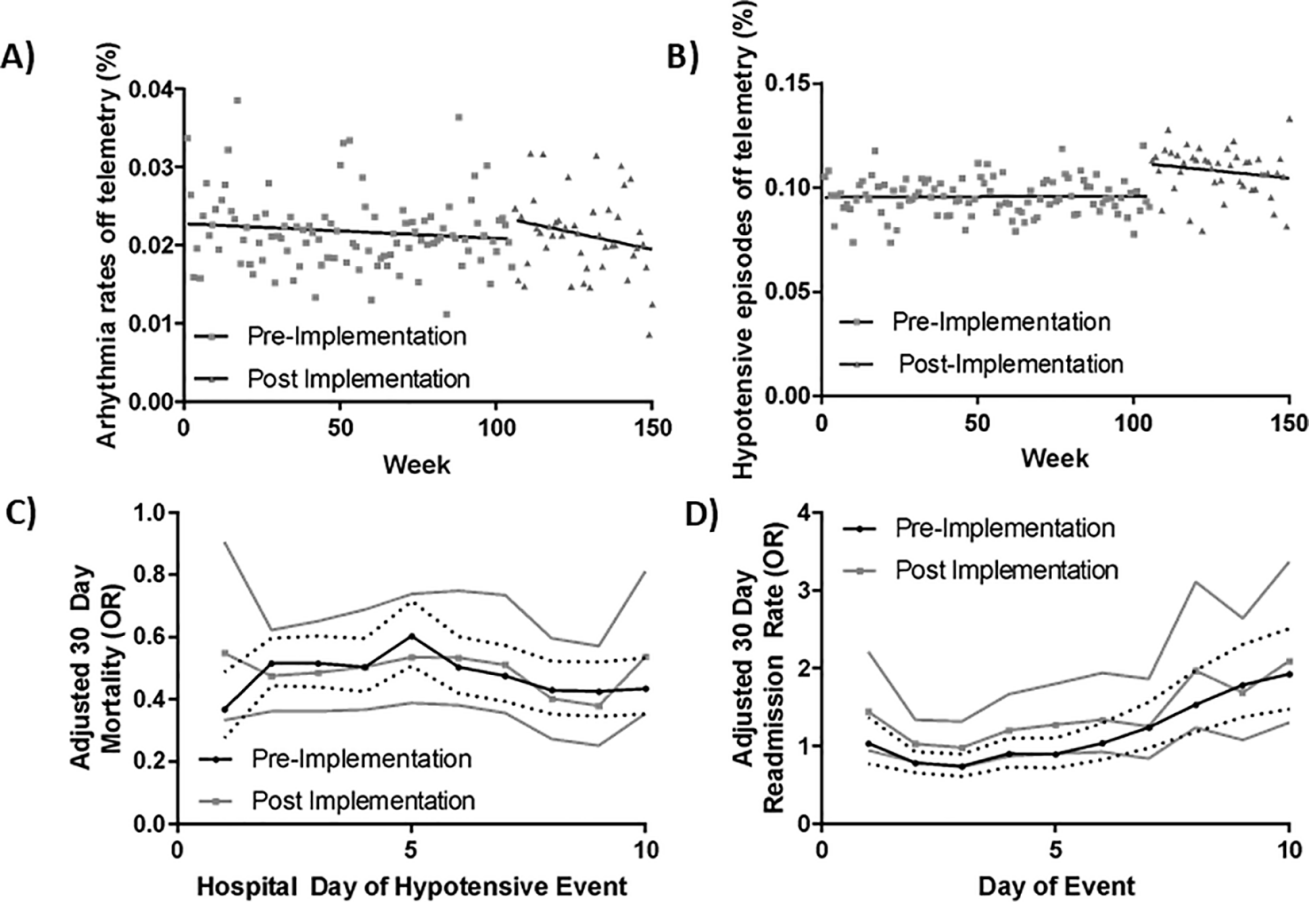
Hemodynamic events off continuous cardiac monitoring. Unadjusted rate and linear trend pre- and post-intervention for hemodynamic events including: cardiac tachyarrhythmia or bradyarrhythmia **(A)** and hypotension **(B)**. For patients with a hypotensive event, the association of that even being off continuous cardiac monitoring and risk adjusted 30-day all-cause mortality **(C)** and 30-day all-cause readmissions **(D)** for a given hospital day when the event occurred.

We investigated hemodynamic event capture rates before and after by the CDS implementation (Table 2). Observed bradycardic events were lower post implementation (odds ratio 0.7504, 95% CI 0.579 to 0.972, p = 0.03) tachycardic events were similar (odds ratio 1.0606, 95% CI 0.920 to 1.223, p = 0.42), and CDS implementation was not associated with increases in the absolute event or the likelihood the event would occur off continuous cardiac monitoring (odds ratio 1.1620, 95% CI 0.995 to 1.357, p = 0.06). However, we did find that the rate of hypotension off continuous cardiac monitoring occurred at a higher rate post implementation, which remained robust when controlling for temporal trends (odds ratio 1.2072, 95% CI 1.122 to 1.299, p < 0.001) (Fig 2 and Table 2).

When adjusting for patient age, race, chronic comorbidities, and day of the hypotensive event, on average, hypotensive episodes off continuous cardiac monitoring were associated with lower risk of death both pre (OR 0.37 95% CI 0.27 to 0.49, p < 0.001 for Day 1) and post (OR 0.26 95% CI 0.181 to 0.375, p < 0.001) and similar rates of 30-day readmission (1.03 95% CI 0.77 to 1.38, p = 0.482). There was no significant interaction between implementation and the risk of death or readmission for patients who had a hypotensive event off continuous cardiac monitoring.

Our findings were robust to excluding ICU admissions (where continuous cardiac monitoring represented standard of care and may not necessarily be ordered). Similar results were observed for white and BIPOC populations, and when excluding patients admitted with COVID-19 (S11 Table in S1 File and S2 and S3 Figs in S1 File). Similar changes were observed across individual community and tertiary care hospitals.

## Discussion

While continuous cardiac monitoring is an important tool, its overuse can potentially decrease efficiency of care, increase health care spending, and negatively impact patient experience. Broad and consistent adoption of AHA continuous cardiac monitoring practice standards remain problematic which may, in part, be due to lack of research on implementation strategies that support the use of these standards. Using the strategy of an EHR embedded CDS which incorporated AHA practice standards, we found through this study that continuous cardiac monitoring was significantly reduced without worsening clinical outcomes.

While ordering rates of continuous cardiac monitoring decreased, ordering that fell outside of practice standards remained common: at least 17.7% of initial continuous cardiac monitoring orders, and 29% of all continuous cardiac monitoring days exceeded duration of practice standards after implementation. We found that most non-cardiac indications related to potential critical illness among a broad range of diagnoses including sepsis, respiratory failure, and acute blood loss. These results may imply that physicians feel that continuous cardiac monitoring can help more rapidly respond to patients who may decompensate. Similarly, we explored the reasons for continuous cardiac monitoring orders and continuation of monitoring, finding that they were limited to a particularly high-risk patient population. This implies a significant proportion of continuous cardiac monitoring ordering outside of practice standards is likely intentional, rather than forgotten discontinuation or provider preference. We have more fully investigated this variation in provider adherence to AHA guidelines, which has been published previously [20]. The AHA practice standards specifically highlight the lack of evidence for or against continuous cardiac monitoring use for patients who are non-critically ill, but at risk for hemodynamic instability. As risk of decompensation was not a component of our CDS, we found that implementation was associated with a higher rate of hypotension occurring off continuous cardiac monitoring.

We found that, on average, adjusted outcomes for a patient who was hypotensive were better when off continuous cardiac monitoring, likely reflecting the overall lower risk of the patient population. We found no evidence that continuous cardiac monitoring was associated with improved outcomes when patients became unstable. Moreover, the increase in occurrence with these episodes was not associated with any observable negative consequences in efficiency of care or subsequent outcomes. These findings suggest that full continuous cardiac monitoring may be effectively replaced by continuous heart rate monitoring when the underlying rhythm is less important than high resolution hemodynamic monitoring.

Unlike other studies, we did not observe a reduction in continuous cardiac monitoring duration despite an interruptive BPA [4,21,22]. We also did not observe reductions in length of stay and ED wait times for a continuous cardiac monitoring bed. This may be related to the more flexible approach toward implementing AHA practice standards compared to other studies. We did not mandate AHA adherence; rather, the ordering process allowed providers to use an “Other” category for non-cardiac indications. We also did not mandate discontinuation, only prompting a reminder. Automating continuous cardiac monitoring discontinuation may further reduce utilization, but would need to be balanced against patient safety, reductions in provider agency, and provider experience of having to re-order continuous cardiac monitoring in a high-risk population which lacks evidence-based practice standards. Overall, this flexibility has the potential to minimize adverse events while reducing telemetry costs. Eliminating monitoring on nonindicated kays could save a minimum of $53 per patient day.(6) In our study, we estimate that averaged over the duration of the study, approximately $17 in telemetry costs were saved per patient, totaling over $833,00 over the course of the study.

Additionally, this work highlights the importance of indication reporting for evaluating continuous cardiac monitoring use [1]. Creation of a CDS tool within the EHR allows for precise association between indication for continuous cardiac monitoring and its appropriateness. Our CDS mandated an indication which allowed for higher quality of data and for in-depth evaluation. Pre-implementation indication reporting rates were well below 40%, preventing direct comparisons across individual indications. While public health campaigns such as *Choosing Wisely* [11] focus on preventing ungoverned continuous cardiac monitoring duration, many successful efforts have included indication documentation as part of the intervention [10,23]. Because indication reporting is necessary for evaluating and monitoring utilization that falls outside of AHA practice standards, indication documentation for continuous cardiac monitoring should be considered as a practice standard.

### Limitations

Our study has limitations that may impact generalization. First, as a quasi-experimental study from a single hospital system with a pre-post analysis, analysis is potentially confounded by seasonality, other ongoing interventions in a large health system, capacity and staffing limitations that could impact telemetry decision making, and COVID-19. Notably, the intervention began during the COVID-19 omicron wave, complicating analysis. Second, analysis of this study occurred 10 months after initiation. This relatively short duration, therefore, limits the ability to determine long-term practice change in the health system. However, we did see adherence rates increase with time, suggesting that providers’ adoption of the intervention improved. The lack of high-quality indication reporting prior to the intervention also prevents several interesting analyses. For example, we cannot determine if the intervention specifically reduced continuous cardiac monitoring use among patients at low risk for an arrhythmia. We additionally cannot measure the degree of AHA non-adherence prior to the intervention with a high degree of confidence.

Finally, while analysis of patients with hemodynamic events finds no association between active continuous cardiac monitoring and subsequent outcomes, additional studies are needed to confirm the findings. For example, we did not specifically compare continuous cardiac monitoring with other continuous heart rate monitoring devices, such as a pulse-oximeter. Our results do not suggest that patients at risk for instability should not be monitored, merely that the benefit of continuous cardiac monitoring may be replicated using other technology that focuses on heart rate or frequent nursing checks. It may be that continuous heart rate monitoring is non-inferior or superior to continuous cardiac monitoring in potentially unstable patients, but this warrants further investigation and is an immediate next step of this study.

## Conclusions

Integrating AHA practice standards into continuous cardiac monitoring ordering into EHR tools reduced cardiac monitoring without worsening patient safety. Nevertheless, monitoring outside of AHA practice standards persisted and was primarily focused on monitoring for potential hemodynamic instability. Monitoring patients with potential instability was reduced by the intervention, yet was still common, resulting in increased hemodynamic events off monitoring. This was not associated with worse outcomes, suggesting continuous cardiac monitoring may not be necessary in this population beyond continuous heart rate monitoring which can be done less invasively.

## Supporting information

S1 File **S1 Fig**. Trends in patient outcomes before and after implementation of telemetry CDS including (A) Length of stay, (B) In hospital Mortality, and (C) 30-day readmission rate demonstrate no worsening in hospital utilization or outcomes with decreased telemetry monitoring. **S2 Fig**. Sensitivity analysis of primary outcomes adjusting for COVID-19. (A) Total Covid-19 hospitalization volume per week from the start of data collection demonstrates the omicron surge which coincided with the implementation date. (B) Trends in total telemetry days, (C) order rate, and (D) in hospital mortality pre and post implementation demonstrating our findings were not related to changes in COVID-19 cases. **S3 Fig**. Equity assessment of the CDS implementation. On average we find that non-white patients were less likely to receive telemetry monitoring both in terms of duration (A) and order rate (B). Impact of the intervention did not vary by race. Indication reporting was slightly lower among non-whites pre-implementation (C). The intervention reduced that disparity. **S4 Table**. Categorization of AHA guidelines within the CDS and recommended durations. **S5 Table**. Impact of CDS on unadjusted telemetry ordering, hemodynamic event rate, and patient outcomes. **S6 Table**. Total and excess telemetry days by general indication post implementation. **S7 Table**. Comparison outcomes for patients without telemetry, guidelines concordant duration, and excess telemetry. **S8 Table**. Categories of reported Non-cardiac indications for telemetry ordering. **S9 Table**. Comparison of outcomes and measures for patients admitted with no telemetry, initial indication was consistent with AHA guidelines or initial indication was noted as “Other”. **S10 Table**. Interrupted time series analysis of primary and secondary measures excluding patients who were admitted or transferred to the ICU. **S11 Table**. Interrupted time series analysis of primary and secondary measures excluding patients who were admitted with COVID-19.
(DOCX)
