# Supplementary material for: Aligning cardiac monitoring with American Heart Association Guidelines: Impact on utilization, hemodynamic monitoring, and outcomes
Source: PLoS One. 2026 Jan 30;21(1):e0338943. doi: 10.1371/journal.pone.0338943 (PMC12858011; doi:10.1371/journal.pone.0338943)
Supplement: S1 File — S1 Fig. Trends in patient outcomes before and after implementation of telemetry CDS including (A) Length of stay, (B) In hospital Mortality, and (C) 30-day readmission rate demonstrate no worsening in hospital utilization or outcomes with decreased telemetry monitoring. S2 Fig. Sensitivity analysis of primary outcomes adjusting for COVID-19. (A) Total Covid-19 hospitalization volume per week from the start of data collection demonstrates the omicron surge which coincided with the implementation date. (B) Trends in total telemetry days, (C) order rate, and (D) in hospital mortality pre and post implementation demonstrating our findings were not related to changes in COVID-19 cases. S3 Fig. Equity assessment of the CDS implementation. On average we find that non-white patients were less likely to receive telemetry monitoring both in terms of duration (A) and order rate (B). Impact of the intervention did not vary by race. Indication reporting was slightly lower among non-whites pre-implementation (C). The intervention reduced that disparity. S4 Table. Categorization of AHA guidelines within the CDS and recommended durations. S5 Table. Impact of CDS on unadjusted telemetry ordering, hemodynamic event rate, and patient outcomes. S6 Table. Total and excess telemetry days by general indication post implementation. S7 Table. Comparison outcomes for patients without telemetry, guidelines concordant duration, and excess telemetry. S8 Table. Categories of reported Non-cardiac indications for telemetry ordering. S9 Table. Comparison of outcomes and measures for patients admitted with no telemetry, initial indication was consistent with AHA guidelines or initial indication was noted as “Other”. S10 Table. Interrupted time series analysis of primary and secondary measures excluding patients who were admitted or transferred to the ICU. S11 Table. Interrupted time series analysis of primary and secondary measures excluding patients who were admitted with COVID-19. (DOCX) [file pone.0338943.s001.docx]

**S1 Figure:** Trends in patient outcomes before and after implementation of telemetry CDS including (A) Length of stay, (B) In hospital Mortality, and (C) 30-day readmission rate demonstrate no worsening in hospital utilization or outcomes with decreased telemetry monitoring.


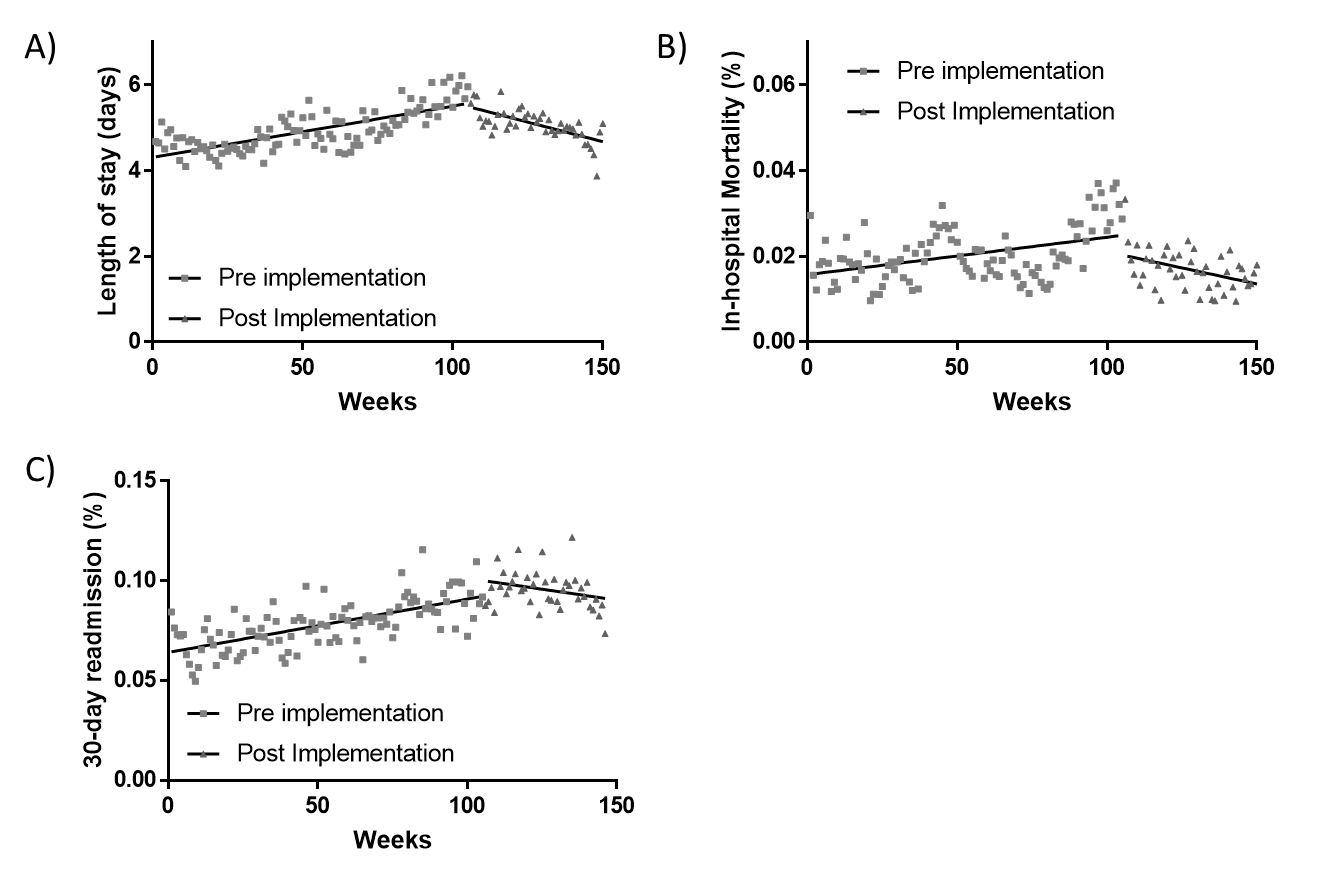


**S2 Figure:** Sensitivity analysis of primary outcomes adjusting for COVID-19. (A) Total Covid-19 hospitalization volume per week from the start of data collection demonstrates the omicron surge which coincided with the implementation date. (B) Trends in total telemetry days, (C) order rate, and (D) in hospital mortality pre and post implementation demonstrating our findings were not related to changes in COVID-19 cases.


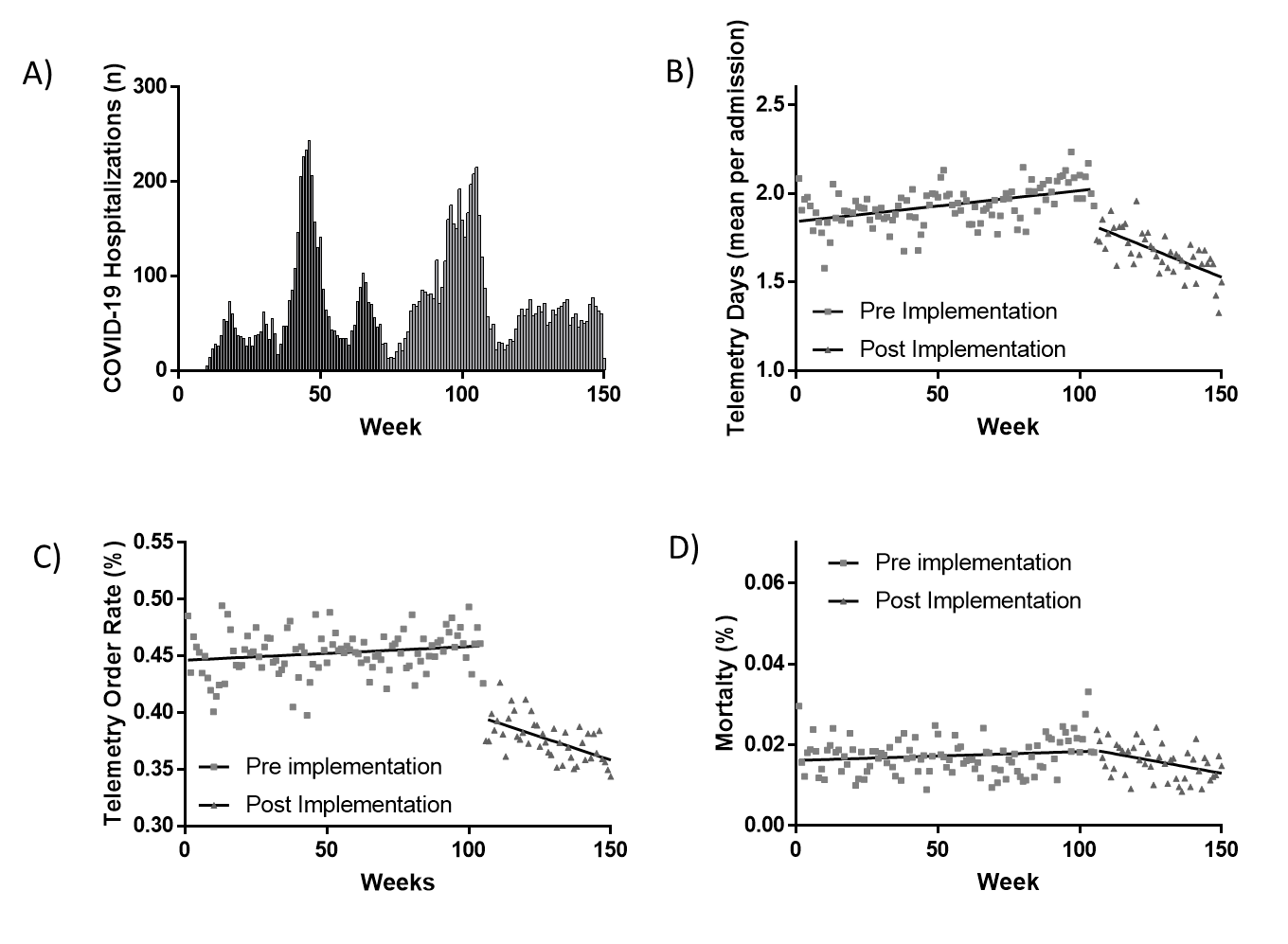


**S3 Figure:** Equity assessment of the CDS implementation. On average we find that non-white patients were less likely to receive telemetry monitoring both in terms of duration (A) and order rate (B). Impact of the intervention did not vary by race. Indication reporting was slightly lower among non-whites pre-implementation (C). The intervention reduced that disparity.


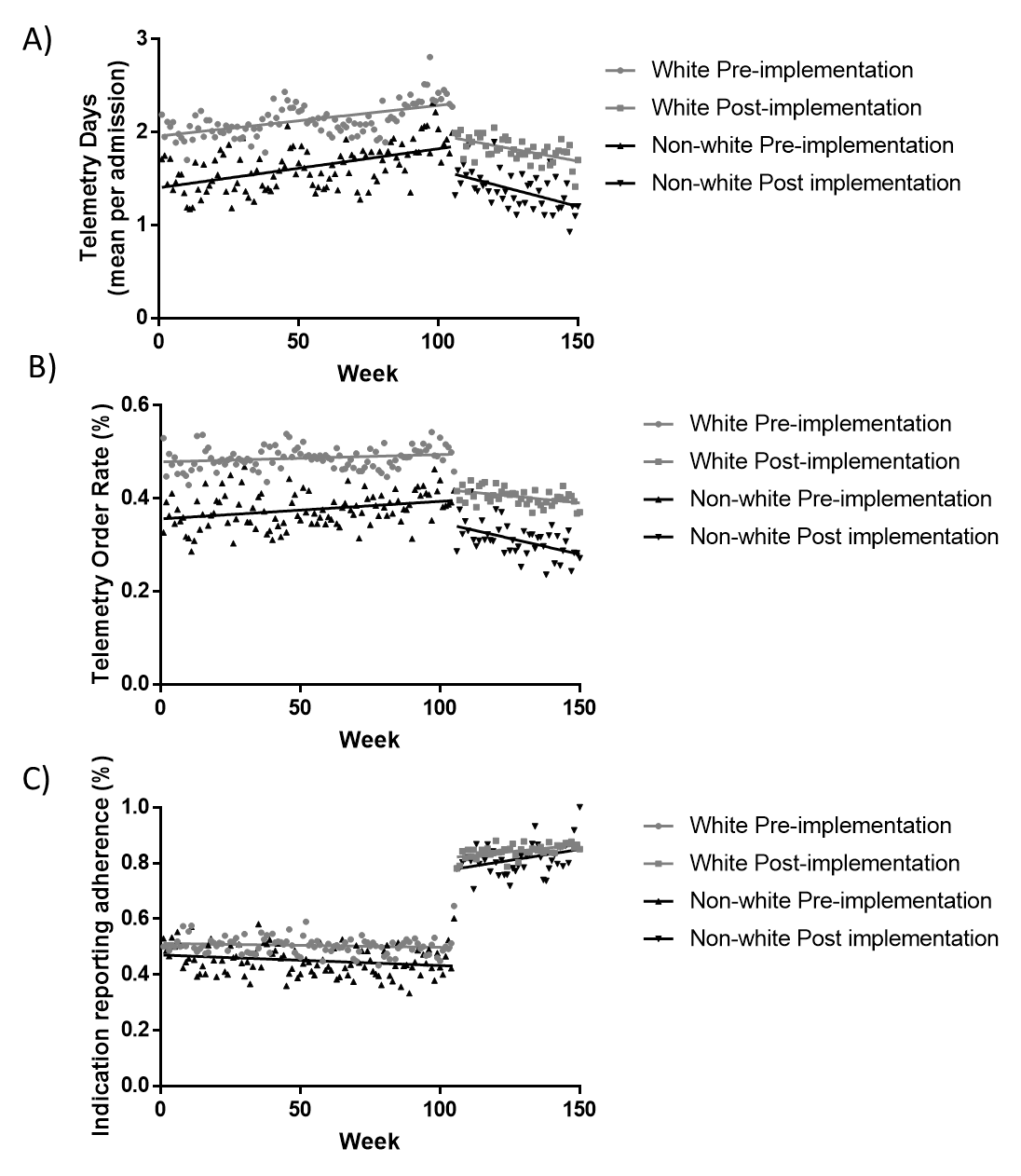


**S4 Table:** Categorization of AHA guidelines within the CDS and recommended durations

| General Indication | Indication | Duration (hours) |
| --- | --- | --- |
| Cardiac | AMI (NSTEMI/ STEMI) | 48 |
|  | Acute decompensated heart failure (48 hours) | 48 |
|  | Bradycardias | 48 |
|  | Chest pain/ ACS rule out | 24 |
|  | Infective endocarditis - additional guidance recommending until clinically stable | 48 |
|  | QTc prolonging medication | 48 |
|  | Syncope- high cardiac risk | 48 |
|  | Syncope- low cardiac risk | 48 |
|  | Tachyarrhythmias, acute | 48 |
| Cardiac Procedure | Open heart surgery | 72 |
|  | Post- EP procedure | 48 |
|  | Post- ICD or pacemaker placement | 48 |
|  | Post- PCI/ percutaneous cardiac intervention | 24 |
|  | Post- PCI/Angiogram | 24 |
|  | Transcatheter structural interventions | 24 |
| Medical | Drug overdose | 24 |
|  | Electrolyte Imbalance - Magnesium <1.3 mg/ml; Potassium < =2.8 or > 5.5 mg/ml | 24 |
|  | Stroke, acute | 48 |
| Procedural area | Procedural area | 24 |
| ICU | ICU | N/A |
| Other | Other | 24 |
| Missing/Non-specified | Non-Adherent to Indication Reporting | N/A |

**S5 Table:** Impact of CDS on unadjusted telemetry ordering, hemodynamic event rate, and patient outcomes.

|  |  | Intercept | | | Slope | | |
| --- | --- | --- | --- | --- | --- | --- | --- |
|  | Measure | Treatment Effect* | 95% CI | p-value | Treatment Effect* | 95% CI | p-value |
| Process | Telemetry Days^1^ | -0.3494 | (-0.412 to -0.287) | <0.001 | -0.0093 | (-0.012 to -0.007) | <0.001 |
|  | Telemetry Ordered^2^ | 0.7420 | (0.709 to 0.777) | <0.001 | 0.9959 | (0.994 to 0.998) | <0.001 |
|  | Indication^2^ | 1.6459 | (1.567 to 1.729) | <0.001 | 0.9987 | (0.997 to 1.001) | 0.1621 |
| Hemodynamic events | Tachycardia^2^ | 0.9764 | (0.849 to 1.123) | 0.7387 | 0.9976 | (0.992 to 1.003) | 0.3866 |
|  | Bradycardia^2^ | 0.7687 | (0.593 to 0.996) | 0.0465 | 1.0064 | (0.997 to 1.016) | 0.2039 |
|  | Arrhythmia off Telemetry^2^ | 1.0520 | (0.903 to 1.226) | 0.5165 | 1.0010 | (0.995 to 1.007) | 0.7392 |
|  | Hypotension^2^ | 1.0032 | (0.946 to 1.064) | 0.9156 | 0.9947 | (0.992 to 0.997) | <0.001 |
|  | Hypotension off Telemetry^2^ | 1.1928 | (1.109 to 1.282) | <0.001 | 0.9979 | (0.995 to 1.001) | 0.1307 |
| Outcomes | In Hospital Mortality^2^ | 0.8466 | (0.720 to 0.996) | 0.0445 | 0.9836 | (0.977 to 0.990) | <0.001 |
|  | 30 Day Readmission^2^ | 1.0689 | (0.990 to 1.154) | 0.0878 | 0.9945 | (0.992 to 0.997) | 0.0002 |
|  | Admission Delay^1^ | 0.3574 | (0.065 to 0.649) | 0.0164 | -0.0147 | (-0.026 to -0.004) | 0.0082 |
|  | Length of Stay^1^ | -0.0822 | (-0.276 to 0.111) | 0.4051 | -0.0270 | (-0.034 to -0.021) | <0.001 |
|  | Log Length of Stay^1^ | -0.0133 | (-0.032 to 0.005) | 0.1588 | -0.0031 | (-0.004 to -0.002) | <0.001 |

Results of an interrupted time series analysis following fitting of a GEE model without age, race, gender, and comorbidities as covariates.

*Treatment effects are mean differences for continuous outcomes (indicated by ^1^) and odds ratios for binary outcomes (indicated by ^2^). Confidence intervals are on the same scale as the treatment effects.

**S6 Table:** Total and excess telemetry days by general indication post implementation.

|  | Total Telemetry Days | Excess Telemetry Days |
| --- | --- | --- |
| Cardiac | 28684 (34.1%) | 8371 (34.3%) |
| Cardiac Procedure | 5061 (6%) | 1160 (4.8%) |
| ICU | 10508 (12.5%) | 3321 (13.6%) |
| Medical | 8966 (10.7%) | 2330 (9.6%) |
| Procedural | 2230 (2.7%) | 0 (0 %) |
| Other | 14903 (17.7%) | 5188 (21.3%) |
| Missing | 13648 (16.2%) | 4489 (18.4%) |
| Total | 84,009 | 34,393 |

**S7 Table:** Comparison outcomes for patients without telemetry, guidelines concordant duration, and excess telemetry

|  | No Telemetry | Guideline concordant Telemetry Duration | Excess Telemetry |
| --- | --- | --- | --- |
| n | 20232 | 12174 | 6600 |
| Length of Stay | 4.3 (6.8) | 4 (4.9) | 10.2 (11.0) |
| Tachycardia | 777 (2.6%) | 181 (1.5%) | 364 (4.0%) |
| Bradycardia | 102 (0.3%) | 134 (1.1%) | 145 (2.2%) |
| Hypotensive | 3553 (11.7%) | 1882 (15.5%) | 2869 (43.5%) |
| ICU Transfer | 1535 (5.1%) | 2276 (18.7%) | 2189 (33.2%) |
| In hospital Mortality | 225 (0.7%) | 317 (2.6%) | 284 (4.3%) |
| 30 Day all-cause Mortality | 642 (2.1%) | 660 (5.4%) | 665 (10.1%) |
| 30 Day all cause Readmission | 1535 (5.1%) | 2276 (18.9%) | 2189 (33.2%) |

**S8 Table:** Categories of reported Non-cardiac indications for telemetry ordering

| Category | N | Telemetry Days, mean (SD) | Excess Days, mean (SD) |
| --- | --- | --- | --- |
| Vital Abnormalities | 280 | 3.89 (2.0) | 1.56 (1.7) |
| DKA | 223 | 3.36 (1.8) | 1.1 (1.7) |
| Alcohol | 176 | 3.75 (2.0) | 1.53 (1.9) |
| GI bleed, anemia | 281 | 3.75 (2.0) | 1.45 (1.8) |
| Level of care | 79 | 4.2 (2.3) | 1.8 (2.1) |
| Neurologic symptoms (not stroke) | 213 | 4.1 (2.1 | 1.7 (1.9) |
| Other | 1103 | 4.1 (2.4) | 1.5 (2.0) |
| Pulmonary Embolism | 108 | 3.7 (2.1) | 1.4 (1.8) |
| Respiratory Failure | 387 | 4.4 (2.5) | 1.9 (2.0) |
| Sepsis | 336 | 4.3 (2.5) | 1.8 (2.2) |
| Surgery | 285 | 2.9 (1.8) | 0.6 (1.3) |
| Trauma | 74 | 3.4 (2.1) | 1.2 (2.0) |
| Total | 3545 | 3.9 (2.2) | 1.5 (1.9) |

**S9 Table:** Comparison of outcomes and measures for patients admitted with no telemetry, initial indication was consistent with AHA guidelines or initial indication was noted as “Other”

|  | No Telemetry | AHA concordant indication | Non-Cardiac Telemetry |
| --- | --- | --- | --- |
| n | 20232 | 12184 | 3545 |
| Length of Stay | 4.3 (6.8) | 6.3 (8.4) | 6.4 (7.4) |
| Tachycardia | 777 (2.6%) | 241 (2.0%) | 91 (2.6%) |
| Bradycardia | 102 (0.3%) | 210 (1.7%) | 46 (1.3%) |
| Hypotensive | 3553 (11.7%) | 3242 (26.6%) | 845 (23.8%) |
| ICU Transfer | 1535 (5.1%) | 3367 (27.6%) | 573 (16.6%) |
| In hospital Mortality | 225 (0.7%) | 424 (34.8%) | 100 (2.8%) |
| 30 Day all-cause Mortality | 642 (2.1%) | 925 (7.6%) | 228 (6.4%) |
| 30 Day all-cause Readmission | 1535 (5.1%) | 1371 (11.3%) | 418 (11.8%) |

**S10 Table:** Interrupted time series analysis of primary and secondary measures excluding patients who were admitted or transferred to the ICU.

|  |  | Intercept | | | Slope | | |
| --- | --- | --- | --- | --- | --- | --- | --- |
|  | Measure | Treatment Effect* | 95% CI | p-value | Treatment Effect* | 95% CI | p-value |
| Process | Telemetry Days^1^ | -0.3907 | (-0.446 to -0.336) | <0.001 | -0.0082 | (-0.010 to -0.006) | <0.001 |
|  | Telemetry Ordered^2^ | 0.6274 | (0.592 to 0.663) | <0.001 | 0.9953 | (0.993 to 0.997) | <0.001 |
|  | Indication^2^ | 1.4038 | (1.322 to 1.491) | <0.001 | 0.9987 | (0.997 to 1.001) | 0.2673 |
| Hemodynamic events | Tachycardia^2^ | 0.9808 | (0.731 to 1.315) | 0.8966 | 0.9969 | (0.986 to 1.008) | 0.5938 |
|  | Bradycardia^2^ | 0.9197 | (0.654 to 1.293) | 0.6301 | 1.0077 | (0.995 to 1.021) | 0.2313 |
|  | Arrhythmia off Telemetry^2^ | 1.2264 | (0.921 to 1.632) | 0.1619 | 1.0042 | (0.994 to 1.015) | 0.4433 |
|  | Hypotension^2^ | 0.9973 | (0.926 to 1.074) | 0.943 | 0.9941 | (0.991 to 0.997) | <0.001 |
|  | Hypotension off Telemetry^2^ | 1.2221 | (1.120 to 1.333) | <0.001 | 0.9975 | (0.994 to 1.001) | 0.1394 |
| Outcomes | In Hospital Mortality^2^ | 0.8137 | (0.630 to 1.051) | 0.1137 | 0.9802 | (0.970 to 0.991) | <0.001 |
|  | 30 Day Readmission^2^ | 1.0506 | (0.964 to 1.145) | 0.2584 | 0.9947 | (0.992 to 0.998) | 0.0017 |
|  | Admission Delay^1^ | 0.2777 | (-0.048 to 0.604) | 0.0948 | -0.0115 | (-0.024 to 0.001) | 0.0621 |
|  | Length of Stay^1^ | 0.0506 | (-0.079 to 0.180) | 0.4445 | -0.0136 | (-0.018 to -0.009) | <0.001 |
|  | Log Length of Stay^1^ | -0.0067 | (-0.024 to 0.010) | 0.4381 | -0.0023 | (-0.003 to -0.002) | <0.001 |

*Treatment effects are mean differences for continuous outcomes (indicated by ^1^) and odds ratios for binary outcomes (indicated by ^2^). Confidence intervals are on the same scale as the treatment effects.

**S11 Table:** Interrupted time series analysis of primary and secondary measures excluding patients who were admitted with COVID-19.

|  |  | Intercept | | | Slope | | |
| --- | --- | --- | --- | --- | --- | --- | --- |
|  | Measure | Treatment Effect* | 95% CI | p-value | Treatment Effect* | 95% CI | p-value |
| Process | Telemetry Days^1^ | -0.2647 | (-0.324 to -0.205) | <0.001 | -0.0058 | (-0.008 to -0.004) | <0.001 |
|  | Telemetry Ordered^2^ | 0.6936 | (0.659 to 0.731) | <0.001 | 0.9968 | (0.995 to 0.999) | 0.0015 |
|  | Indication^2^ | 1.7853 | (1.688 to 1.888) | <0.001 | 0.9999 | (0.998 to 1.002) | 0.9494 |
| Hemodynamic events | Tachycardia^2^ | 1.0857 | (0.937 to 1.258) | 0.2742 | 0.9941 | (0.989 to 1.000) | 0.0432 |
|  | Bradycardia^2^ | 0.9992 | (0.750 to 1.331) | 0.9956 | 1.0119 | (1.001 to 1.023) | 0.0285 |
|  | Arrhythmia off Telemetry^2^ | 1.1942 | (1.017 to 1.403) | 0.0308 | 0.9970 | (0.991 to 1.003) | 0.3362 |
|  | Hypotension^2^ | 1.0461 | (0.983 to 1.114) | 0.1575 | 0.9956 | (0.993 to 0.998) | <0.001 |
|  | Hypotension off Telemetry^2^ | 1.1955 | (1.108 to 1.291) | <0.001 | 0.9972 | (0.994 to 1.000) | 0.0572 |
| Outcomes | In Hospital Mortality^2^ | 1.0198 | (0.852 to 1.220) | 0.8306 | 0.9911 | (0.984 to 0.998) | 0.0146 |
|  | 30 Day Readmission^2^ | 1.0519 | (0.969 to 1.142) | 0.2261 | 0.9951 | (0.992 to 0.998) | 0.0019 |
|  | Admission Delay^1^ | 0.2564 | (-0.042 to 0.555) | 0.0924 | -0.0135 | (-0.025 to -0.002) | 0.017 |
|  | Length of Stay^1^ | 0.1534 | (-0.035 to 0.342) | 0.1111 | -0.0191 | (-0.026 to -0.013) | <0.001 |
|  | Log Length of Stay^1^ | 0.0198 | (0.001 to 0.038) | 0.0354 | -0.0019 | (-0.003 to -0.001) | <0.001 |

*Treatment effects are mean differences for continuous outcomes (indicated by ^1^) and odds ratios for binary outcomes (indicated by ^2^). Confidence intervals are on the same scale as the treatment effects.
